# Negative effects of prolonged dietary restriction on male mating effort: nuptial gifts as honest indicators of long-term male condition

**DOI:** 10.1038/srep21846

**Published:** 2016-02-24

**Authors:** Renato C. Macedo-Rego, Luiz Ernesto Costa-Schmidt, Eduardo S. A. Santos, Glauco Machado

**Affiliations:** 1 Programa de Pós-graduação em Ecologia, Instituto de Biociências, Universidade de São Paulo, Rua do Matão, trav. 14, n° 321, São Paulo, SP, 05508-090, Brazil; 2 LAGE do Departamento de Ecologia, Instituto de Biociências, Universidade de São Paulo, Rua do Matão, trav. 14, n° 321, São Paulo, SP, 05508-090, Brazil; 3 Programa de Pós-graduação em Biologia, Universidade do Vale do Rio dos Sinos, Avenida Unisinos, 950, São Leopoldo, RS, 93022-000, Brazil; 4 Laboratorio de Biología Reproductiva y Evolución, Universidad Nacional de Córdoba, Instituto de Diversidad y Ecología Animal (IDEA – CONICET), Avenida Vélez Sarsfield, 299, Córdoba Capital, CP: 5000, Argentina; 5 Departamento de Zoologia, Instituto de Biociências, Universidade de São Paulo, Rua do Matão, trav. 14, n° 321, São Paulo, SP, 05508-090, Brazil

## Abstract

The handicap principle proposes that sexual signals must be costly to be honest. Honesty may be maintained by the costs paid by honest signallers or by the potential costs of cheating. In the latter, handicaps should emerge as a consequence of specific biological constraints, such as life-history trade-offs. Nuptial prey-giving arthropods are good systems to investigate the honesty of sexual signals taking into account trade-offs between self-maintenance and mating effort. We experimentally evaluated if prolonged food shortage during early adulthood imposes long-term negative effects on gift construction by males of the spider *Paratrechalea ornata*. We also evaluated whether a burst of food availability improved body condition of poorly fed males, increasing their frequency of gift construction. Poorly fed males hardly constructed gifts, even after a marked increase in feeding rate, which clearly improved their body condition. Moreover, initially poorly fed males that latter received high food intake constructed lighter gifts than continuously well fed males. The long-term effects of prolonged dietary restriction on male propensity to construct a gift and on the size of this gift may increase the honesty of this sexually selected signal. From the female’s perspective the offer of a gift may bring information on male quality.

A fundamental question in the debate on honesty of animal communication is how the correlation between the observed signal and the non-observable quality is achieved^1^. Although honesty can be generated by several mechanisms, the handicap principle, which proposes that signals must be costly to be honest^2^, has received most attention by researchers interested in sexual selection (review in^3^). Several authors, however, argue that honesty is not maintained by the costs paid by the honest signallers, but by the potential costs of cheating^1^. According to this view, the existence of a handicap is not a theoretical necessity, but rather the result of specific biological constraints that may result, for instance, from resource-based trade-offs between self-maintenance and male mating effort (reviewed in^1^). However, empirical evidence for a direct physiological link between self-maintenance (e.g., immunocompetence) and a sexually selected male trait (e.g., ornamentation) is still scarce and restricted to few animal groups^4^.

Nuptial prey giving arthropods are good model systems to investigate the honesty of male sexual signals because of a particular trade-off between self-maintenance and male mating effort in the group. After capturing a prey, a male can allocate it to self-maintenance or to the construction of the nuptial gift, a sexually selected trait that is known to entice females to copulate and to increase both sperm transfer and the number of eggs sired by the male^5^,^6^,^7^,^8^. However, contrary to other systems in which trade-offs between self-maintenance and male mating effort involve complex metabolic pathways or hormonal feedbacks, here the very same resource (i.e., a prey) can be used either as food to self-maintenance or as a gift, which is a key component of male mating effort^5^,^9^. Therefore, male investment in nuptial gifts clearly creates a compromise with investment in body condition, which is directly linked to food intake^10^. Accordingly, this compromise should represent a case in which honesty is maintained by exerting an appreciable cost that hinders cheating.

If the nuptial gift indeed represents an honest signal, the decision of whether to allocate a prey to self-maintenance or reproduction should be conditional on the nutritional state of a male. Experimental studies with the prey giving spider *Pisaura mirabilis* (Pisauridae) indicate that satiated males construct nuptial gifts more frequently and invest more energy depositing a thick layer of silk on these gifts than starved males in the presence of female cues^11^. These findings suggest that dietary restriction during the adult phase imposes a short-term negative effect on the gift construction behaviour. In another prey giving spider, *Paratrechalea ornata* (Trechaleidae), poorly fed males deposit less silk when constructing a gift than well fed males, but no information on the frequency of gift construction is available^12^. In both spider species, the effect of the diet on the quality of the gift was evaluated only once during the males’ lifetime, but the negative effect of dietary restriction in spiders may also have long-term implications for the individuals. For instance, when females of *Lycosa tarantula* (Lycosidae) experience food limitation during the immature stages, they show an increased rate of sexual cannibalism as adults, probably as a compensatory response to prey shortage in the past^13^. Cannibalistic females have higher rates of reproduction and produce higher-quality offspring than non-cannibalistic females^14^. In prey giving spiders, though, a long-term propensity to feed on the prey could severely reduce the fitness of starved males because female mating decisions may be based on the presence or quality of the gift^5^,^6^,^15^ (but see^16^,^17^). Consequently, gift giving could act as an honest signal of a male’s long-term nutritional condition, as nutritionally restricted males would be unable to cheat and produce the sexual signal.

Here we performed an experiment to evaluate if prolonged dietary restriction during the early adult phase of males imposes long-term negative effects on gift construction using the spider *P. ornata* as study system. Courtship in this species involves the transfer of a prey wrapped in silk from the male to the female^9^. Nuptial prey giving is interpreted as a key component of the male mating effort^6^, and its expression is triggered by female cues^18^. By assessing males in three different moments of their adult life, we first tested the hypothesis that poorly fed males are less prone to construct a gift than well fed males. The results of this first test provide information on whether gift construction honestly advertises current male condition. Then we tested whether a burst of food availability to poorly fed males increases their body condition, and consequently increases their frequency of gift construction. Should poorly fed males increase body condition, but are still unable to construct a nuptial gift, we have evidence that gift construction is also a reliable indicator of long-term male condition.

## Methods

### Experimental design

**Figure 1:**
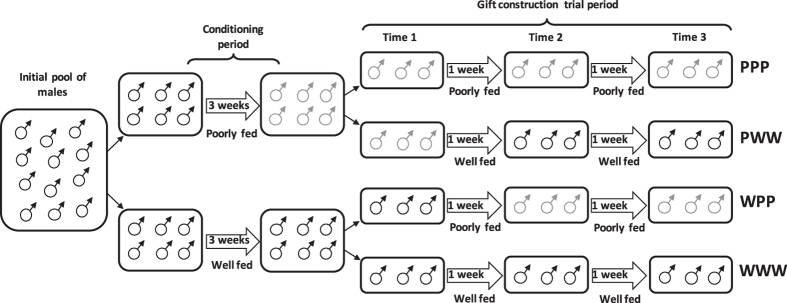
Scheme of the experimental design (the number of males within each box does not represent the actual sample size). The initial pool of males was randomly divided into two groups that were either well fed or poorly fed during three consecutive weeks. After this conditioning period, each group was split into two experimental groups in which males were either poorly or well fed in the following two weeks. Based on the feeding regime experienced by the males over the course of the entire experiment, four groups were established: poorly-poorly-poorly fed (PPP), poorly-well-well (PWW), well-poorly-poorly (WPP), and well-well-well (WWW). Gift construction by the males in each experimental group was evaluated in three moments along the experiment: Times 1, 2 and 3.

We collected subadult males and females of *P. ornata* in the Pedra de Amolar river (29°32′20″ S, 50°14′46″ W), state of Rio Grande do Sul, Brazil, in February 2013 and February 2014. We brought all individuals to the laboratory where we reared them in individual plastic vials (8 cm high x 6 cm in diameter) covered with a soft textile net. Each vial contained a small wood stick as a perch for the spider and a piece of wetted cotton to maintain humidity. Temperature in the laboratory ranged from 21 °C to 25 °C and the light:dark cycle was 12:12 h. We fed all subadults three times per week with laboratory-reared cockroach nymphs (ca. 5 mm). Approximately 24 h after their last moult, we weighed spiders using a digital balance (to the nearest 0.0001 g) to record their body mass and photographed each male to measure cephalothorax width in the software Image*J* (US National Institutes of Health, Bethesda, MD, http://imagej.nih.gov/ij). Then, we randomly divided virgin males into two groups (Fig. 1): (a) poorly fed (n = 45), in which each male received one small cockroach nymph (mean ± SD = 6.90 ± 2.10 mg, which corresponds to 9.73% of mean male body mass) once per week for three consecutive weeks; and (b) well fed (n = 50), in which each male received three small cockroach nymphs once per week for three consecutive weeks. We called ‘conditioning period’ this three-week period before the beginning of the gift construction trial period (Fig. 1). Considering that *P. ornata* males probably require from 5 to 10 days to complete their sexual maturity after the final molt^15^, the 21-day period of conditioning is likely to be long enough even for poorly fed males to be sexually developed.

By the end of the conditioning period, we split each of the initial groups (poorly and well fed) into two experimental groups in which males were either poorly or well fed during the following two weeks (Fig. 1). Therefore, based on the feeding regimes experienced by the males after the conditioning period, four experimental groups were established (Fig. 1): (1) poorly-poorly-poorly (PPP) fed males (n = 22) received one small cockroach nymph once per week for the following two weeks; (2) poorly-well-well (PWW) fed males (n = 23) received three small cockroach nymphs once per week for the following two weeks; (3) well-well-well (WWW) fed males (n = 25) received three small cockroach nymphs once per week for the following two weeks; and (4) well-poorly-poorly (WPP) fed males (n = 25) received just one small cockroach nymph per week for the following two weeks. The total duration of the experiment was five weeks, which corresponds to 30–40% of the males’ adult lifetime in the laboratory (mean ± SD = 94.3 ± 21.6 days, range: 51–121 days, n = 56).

As soon as we divided the males in the four experimental groups described above, we submitted the males to the first trial of gift construction (Time 1; Fig. 1). We placed each male inside a Petri dish (9 cm diameter) with the bottom covered with a filter paper containing the draglines (silk) of one virgin female as a cue for prey gift construction (following^15^,^18^). After 15 min of acclimation, we offered each male a cockroach nymph, and recorded whether the males were holding or not a gift in their chelicerae at the end of a two-hour period. By recording the presence of a gift after two hours we could ensure that males really invested in nuptial gift construction, and not simply wrapped the prey for immediate consumption, as normally occurs in trechaleids^19^. At the end of the trial, males were replaced in their respective vials (without any female cue), where they were allowed to eat the prey or the gift they constructed. The cockroach nymph eaten at this first trial was considered part of the feeding regime of the males in the following phase of the experiment (Fig. 1). Thus, we did not offer another prey for males designated to be poorly fed (WPP and PPP), and for males designated to be well fed (PWW and WWW), we offered two additional nymphs in the day after the gift construction trial.

We performed the procedure described above at the end of the fourth week (Time 2) and at the end of the fifth week (Time 3) (Fig. 1). Before each trial (Times 1 to 3), to ensure that the feeding regime was influencing body condition, we weighed each male using a digital balance. After the trial in Time 3, we collected the gift constructed by each male (if any gift was constructed at all) and preserved it in 70% ethanol. We recorded the dry weight of each gift produced (to the nearest 0.0001 g) to evaluate the proportion of the nymph consumed by each male and to compare prey consumption among experimental groups.

### Statistical analyses

Given that the cephalothorax in *P. ornata* does not change in size or shape after maturity, while total body mass changes according to the feeding regime experienced by the males, we estimated the body condition index of the initial pool of males as the residuals of a regression between body mass and cephalothorax width^20^. This regression approach provides an estimate of body condition that is uncorrelated with body size, and it is a widely used method in studies with spiders^21^,^22^. To evaluate whether the conditioning period generated differences in body condition between well fed and poorly fed males before the onset of the experiment, we performed a linear mixed model in the R package MCMCglmm^23^ using the body condition index as the response variable and group (categorical with two levels: well fed and poorly fed males) and time (categorical with two levels: beginning and end of the conditioning period) as predictor variables. We used male identity as a random factor in the analysis to account for repeated measures of the same individuals, and an Inverse Wishart prior (variance structures: *R* and *G*: *nu* = 0, *V* = 1, and fixed effects: *B*: *mu* = 0 and *V* = I*1e^10^). This model allowed us to infer whether the initial sample of males were similar with regards to body condition at the beginning of the conditioning period, and whether the conditioning period had the desired effect of improving the condition of well-fed males, while decreasing the condition of poorly fed males.

To show the effect of diet on male body condition for each experimental group throughout the experiment, we performed another linear mixed model. In this case, we estimated the residual of each male as the difference between his mass in each time of the experiment and the predicted value (based on his cephalothorax width) by the regression performed in the beginning of the conditioning period. Supposing this regression describes the size vs. mass relationship of males in normal feeding conditions, any deviation from the predicted values indicates that males increased or decreased their body mass according to the feeding regime. Following the procedure of the conditioning period, we used the body condition index as the response variable and the experimental group and time as predictor variables. Again, we used male identity as random factor to account for repeated measures of the same individuals, and an Inverse Wishart prior.

To compare the frequency of gift construction and analyse the influence of experimental group and time on a male’s decision, we performed a generalized linear mixed model in the MCMCglmm package^23^. We used the binomial family function (categorical with logit link function), and the response variable was the data from gift construction (no = 0 and yes = 1). We also used experimental group and time as predictor variables and male identity as a random factor (prior: *B*: *mu* = 0 and *V* = diag(*x*)*(1 + π^2/3^), where *x* is the number of fixed effects in the model; *R*: *V* = 1, fix = 1; *G*: *V* = 1, *nu* = 0.002). Lastly, to compare the mass of gifts constructed by males in the last trial (Time 3), we performed a linear model. We excluded data from PPP, since only one male of this experimental group constructed a gift. As the response variable we used gift dry mass, and as the predictor we used the experimental group. We examined the assumption of homogeneity of variances for all linear models by comparing model fit assuming different variance structures^24^, and performed all statistical tests using the program R^25^.

## Results

### Male body condition

Male body condition was similar between the two male groups at the beginning of the conditioning period (Intercept_Poorly fed mean condition_ = 0.0010, SE = 0.0012, β_Well fed_ = −0.0019, 95%CI = −0.0055 to 0.0013, *p*MCMC = 0.276). After three weeks the feeding regimes generated significant differences in male body condition between the two groups. The body condition of well fed males improved whereas the body condition of poorly fed males decreased during the conditioning period (β_Well fed x time_ = 0.0071, 95%CI 0.0067 to 0.0075, *p*MCMC < 0.001; β_Poorly fed x time_ = −0.0019, 95%CI = −0.0022 to −0.0016, *p*MCMC < 0.001).

**Figure 2:**
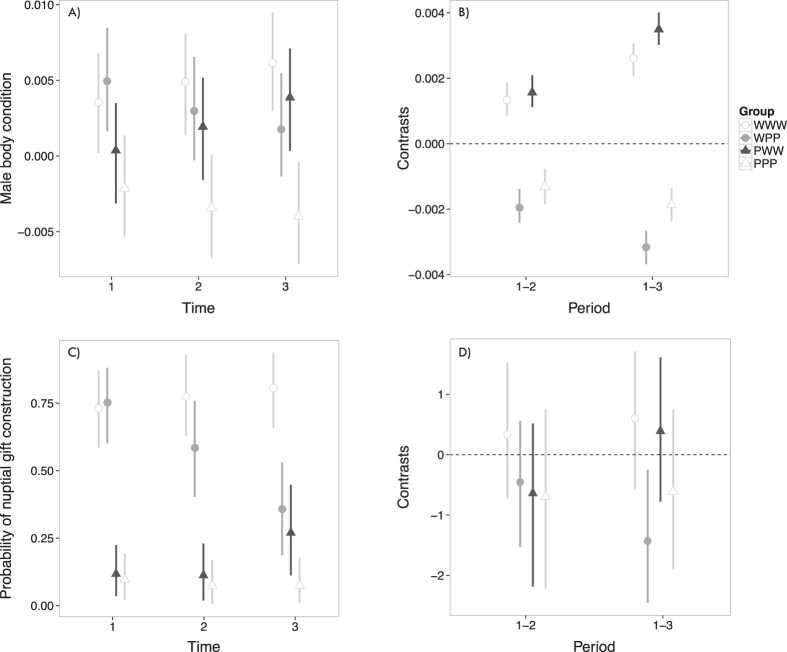
Body condition and probability of nuptial gift construction throughout the experiment. (**A**) Variation in the body condition index (measured as the residuals of a cephalothorax width x body mass regression) of *Paratrechalea ornata* males over the course of the gift construction trial period. (**B**) Contrasts of body condition index for each experimental group between time periods. (**C**) Probability of nuptial gift construction in each experimental group over the course of the experiments. (**D**) Contrasts of probability of nuptial gift construction for each experimental group between time periods. Symbols indicate mean values in (**A**,**C**) or mean differences in (**B**,**D**). In all panels, vertical bars indicate 95% confidence intervals. In (**B**,**D**), when 95% confidence intervals do not overlap zero, the mean differences are considered statistically significant. PPP = poorly-poorly-poorly fed (n = 22 males), PWW = poorly-well-well fed (n = 23 males), WPP = well-poorly-poorly fed (n = 25 males), and WWW = well-well-well fed (n = 25 males).

The variation in male body condition over the gift construction trial period was explained by an interaction between experimental group and time (Fig. 2A,B). In WPP and PPP, male body condition decreased significantly over time (Table 1, Fig. 2B), whereas male body condition increased significantly from Time 1 to 3 in WWW and PWW (Table 1, Fig. 2B).

### Nuptial gift construction

In Time 1, most of the well fed males (WWW and WPP) constructed a gift, but only a small proportion of poorly fed males (PWW and PPP) did so (Fig. 2C). There is little evidence of a change in the frequency of gift construction between Times 1 and 2, and finally between Times 1 and 3, for all groups, with one exception: in WPP, the frequency of gift construction decreased significantly between Times 1 and 3 (Table 2, Fig. 2C,D). For WWW, PPP, and PWW the interaction between experimental group and time was non-significant (Table 2, Fig. 2D). However, the frequency of gift construction was higher in WWW when compared to the other three groups at the last test (Fig. 2C).

### Mass of the nuptial gift

**Figure 3:**
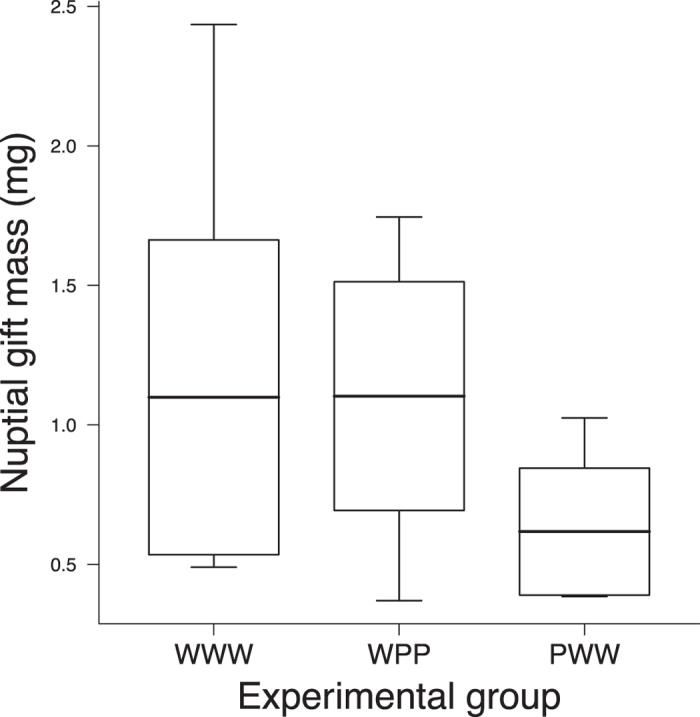
Mass of the nuptial gifts. Comparison of the mass of the gifts constructed by *Paratrechalea ornata* males that experienced different feeding regimes: WWW = well-well-well fed (n = 19), WPP = well-poorly-poorly fed (n = 8) and PWW = poorly-well-well fed (n = 6). The thick horizontal line is the mean, the box is the standard deviation, and the vertical lines indicate the minimum and maximum values in the sample.

The mass of the gifts in the third trial differed among treatments. There was no significant difference between WWW and WPP (t_30_ = 0.021, *p* = 0.984), but the mass of the gift in these two treatments was, on average, significantly heavier than in PWW (t_30_ = 2.10, *p* = 0.044, Fig. 3).

## Discussion

Our results provide evidence of long-term negative effects of prolonged dietary restriction on nuptial gift construction. As expected, most of the *P. ornata* males that starved for three weeks after the final moult did not construct a gift in Time 1. The striking result, however, was that the frequency of gift construction in males that were initially poorly fed but latter received a burst of food intake did not change significantly over the course of Times 2 and 3, despite the fact that male feeding rate increased threefold when compared to constantly poorly fed males. While it is to be expected that constantly poorly fed males always prefer to eat the prey, the same cannot be said about poorly fed males that had their feeding rate increased after Time 1; even after a marked increase in food intake, which clearly improved their body condition, most males did not construct a gift. Therefore, the long-term male response seems to be strongly influenced by the first three weeks of feeding after the final moult, suggesting that starvation imprints a behavioural pattern on males that cannot be easily reverted. This pattern was not observed in initially well fed males that had their feeding rate reduced after Time 1. In this case, both male body condition and frequency of gift construction showed a clear reduction in Times 2 and 3. The different behavioural patterns reported for males of the two groups that changed their feeding regime during the experiment reinforce the notion that the construction of prey gifts is more sensitive to periods of starvation than to periods of food abundance. Besides the difference in the frequency of gift construction, we also found that the mass of the gift was lower in PWW when compared to WWW and WPP (Fig. 3), which provides additional evidence of long-term negative effects of prolonged dietary restriction.

The increase in the ingestion rate under recovery conditions is known as hyperphagia, a behaviour that has already been reported for many vertebrates and invertebrates^26^. We suggest that the high frequency of prey consumption observed during the entire experiment in males that were initially poorly fed but then received high food intake is a hyperphagic response to prey shortage in the past. This behavioural response is analogous to that described for *Lycosa tarantula* females, which show high rates of sexual cannibalism as adults if they experience a prolonged period of starvation during the nymphal stages^13^. The consequences of such a long-term behavioural change for males’ fitness may be profound. In a captive experiment, only 16% of the *P. ornata* males not carrying a gift achieved copulation^15^, indicating that female preference for prey giving males is strong. Therefore, males that starved for long periods after reaching adulthood are expected to have lower mating success when compared to males that had constant access to food. Furthermore, the population of *P. ornata* studied here occurs sintopically with the congeneric prey giving species *P. azul*^9^. Males of *P. ornata* that erroneously court the larger females of *P. azul* are often attacked and consumed^15^. Almost all *P. ornata* males that escaped an attack from *P. azul* females released their gifts, possibly as a tactic to increase their speed and/or to distract the potential predator^15^. Thus, besides the possible mating costs, *P. ornata* males that do not construct a gift may also be more exposed to predation if they erroneously court heterospecific females.

The few males that changed their feeding regime from poorly to well fed and constructed a gift in the last phase of the experiment (Time 3) consumed nearly 91% of the prey before wrapping it. Consequently, the mean mass of the gift in this treatment was nearly 44% lighter than the mean mass of gifts constructed by males that were constantly well fed and males that changed their feeding regime form from well to poorly fed (Fig. 3). It is already known that *P. ornata* males with larger gifts achieve longer copulations, and probably transfer more sperm to their partners^27^. Thus, the size of the gift may increase male fertilization success, especially in a scenario where males face intense sperm competition^28^,^29^. According to this rationale, even if a male that experienced a prolonged starvation period succeeds in constructing a gift and achieves copulation, the intromission will be short, which may restrict the amount of sperm transferred to the female. Moreover, males that offer a small gift show shorter periods between insertions and reduced duration of the “face-to-face” posture^27^, during which females may evaluate male and/or gift quality^12^. Consequently, males that experience a prolonged starvation period and construct a small gift will court the female for less time, which may decrease their fertilization success if females are able to exert some type of cryptic choice. In fact, females of the prey giving spider *Pisaura mirabilis* that accept gifts from males in poor condition do not produce viable offspring^16^. Thus, prolonged dietary restriction reduces both the number and quality of gifts constructed by the males, which leads to long-term fitness costs.

Spiders usually experience food shortage under natural conditions, and show numerous physiological and ecological responses to this important selective pressure^30^,^31^. Our experiment demonstrated that the frequency of gift construction in a prey giving spider is negatively affected by both short- and long-term food shortage, suggesting that gift construction honestly advertises current and past male condition. Therefore, the presence of a gift provides reliable information to females upon which to base the discrimination of possible mating partners because poorly fed males are unable to cheat. According to this rationale, courting males carrying a gift probably did not suffer from regular dietary restriction (equivalent to WWW), whereas courting males without a gift either experienced a long lasting period of starvation in the past (PWW or PPP) or were unable to find food in the last couple of weeks (WPP). From the females’ perspective, males carrying a gift may represent high-quality partners regarding their foraging ability, which in turn may be positively correlated with other condition dependent male attributes, such as immune function^32^ and ejaculate traits^33^. Moreover, given that males that experienced prolonged dietary restriction constructed smaller gifts, females may also use gift size as an indicator of male condition^11^. By selecting males with large gifts, females are not only mating with males in better condition, but they may also acquire more direct benefits from consuming a more substantial meal^8^.

Male traits such as body size, weaponry or ornaments are generally condition dependent in arthropods, and thus are sensitive to food intake during the juvenile period^34^. By experimentally imposing dietary restriction after the males’ final moult to adulthood, we avoided changes to male structural body size or any other morphological trait that could be used by females as a form of mate quality evaluation. Nevertheless, dietary restriction during early adulthood clearly decreased male body condition in *P. ornata*, impairing gift construction by poorly fed males. These poorly fed males allocated most captured prey to self-maintenance, probably to avoid the immediate risk of death by starvation^35^. Altogether our findings suggest that prey gifts are honest signals of male quality as males in poor condition are prevented from cheating^1^. Moreover, the long-term negative effect that dietary restriction has on gift construction reveals that the gift honestly signalizes both current (as in^11^,^12^) and past male condition. Such long-term negative effect may increase the honesty of the signal because a burst of food intake can bring poorly fed males to a better body condition, but it does not increase the frequency of gift construction. Consequently, males that experience prolonged dietary restriction reduce their mating effort, and we predict that they will have lower mating and fertilization success, and will also sire offspring of lower quality.

## Additional Information

**Data availability**: All data used in analyses can be accessed through the figshare repository: https://dx.doi.org/10.6084/m9.figshare.1615032.v1.

**How to cite this article**: Macedo-Rego, R. C. *et al.* Negative effects of prolonged dietary restriction on male mating effort: nuptial gifts as honest indicators of long-term male condition. *Sci. Rep.* **6**, 21846; doi: 10.1038/srep21846 (2016).

## Figures and Tables

**Table 1:** Summary of model estimated contrasts in body condition between the time periods of the experiment with *Paratrechalea ornata* males.

Experimental group	Time	Contrast	Lower 95% CI	Upper 95% CI	pMCMC
PPP	1–2	−0.0013	−0.0018	−0.0008	<0.001
PPP	1–3	−0.0018	−0.0024	−0.0013	<0.001
PWW	1–2	0.0015	0.0011	0.0020	<0.001
PWW	1–3	0.0035	0.0030	0.0040	<0.001
WPP	1–2	−0.0019	−0.0024	−0.0013	<0.001
WPP	1–3	−0.0031	−0.0036	−0.0026	<0.001
WWW	1–2	0.0013	0.0008	0.0018	<0.001
WWW	1–3	0.0026	0.0020	0.0030	<0.001

Experimental groups are PPP: poorly-poorly-poorly fed, PWW: poorly-well-well fed, WPP: well-poorly-poorly fed, and WWW: well-well-well fed males. See details on the experimental design in Fig. 1.

**Table 2:** Summary of model estimated contrasts in the probability of gift construction by *Paratrechalea ornata* males between the time periods of the experiment.

Experimental group	Time	Contrast	Lower 95% CI	Upper 95% CI	pMCMC
PPP	1–2	−0.7978	−2.5762	0.8754	0.382
PPP	1–3	−0.7094	−2.2017	0.8764	0.398
PWW	1–2	−0.6387	−2.1857	0.5174	0.364
PWW	1–3	0.3929	−0.7784	1.6138	0.506
WPP	1–2	−0.4534	−1.5320	0.5621	0.406
WPP	1–3	−1.4286	−2.4556	−0.2504	0.008
WWW	1–2	0.3371	−0.7294	1.5288	0.558
WWW	1–3	0.6030	−0.5658	1.7126	0.328

Experimental groups are PPP: poorly-poorly-poorly fed, PWW: poorly-well-well fed, WPP: well-poorly-poorly fed, and WWW: well-well-well fed males. See details on the experimental design in Fig. 1.
